# Synergic effects of decellularized bone matrix, hydroxyapatite, and extracellular vesicles on repairing of the rabbit mandibular bone defect model

**DOI:** 10.1186/s12967-020-02525-3

**Published:** 2020-09-22

**Authors:** Asrin Emami, Tahereh Talaei-Khozani, Saeid Tavanafar, Nehleh Zareifard, Negar Azarpira, Zahra Vojdani

**Affiliations:** 1 Tissue Engineering Lab, Anatomy Department, Shiraz University of Medical Sciences, Shiraz, Iran; 2 Department of Oral and Maxillofacial Surgery, School of Dentistry, Shiraz University of Medical Sciences, Shiraz, Iran; 3 Transplantation Research Center, Shiraz University of Medical Sciences, Shiraz, Iran

**Keywords:** Decellularized bone, Extracellular vesicle, Hydroxyapatite, Tissue engineering

## Abstract

**Background:**

Extracellular vesicles (ECV) and bone extracellular matrix (ECM) have beneficial effects on the treatment of some pathological conditions. The purpose of this study was to find the synergic effects of decellularized bone (DB) ECM and ECVs on the repair of rabbit.

**Methods:**

The quality of decellularized sheep bones was confirmed by H&E, Hoechst, DNA quantification, immunohistochemistry, histochemical staining, and scanning electron microscopy (SEM). Osteoblast-derived ECVs were evaluated by internalization test, Transmission electron microscopy, Dynamic light scattering, and flow cytometry for CD9, CD63, CD81 markers. The hydrogel containing DB and hydroxyapatite (HA) with or without ECVs was evaluated for osteoblast functions and bone repair both in vitro and in vivo.

**Results:**

The data indicated ECM preservation after decellularization as well as cell depletion. In vitro assessments revealed that mineralization and alkaline phosphatase activity did not improve after treatment of MG63 cells by ECVs, while in vivo morphomatrical estimations showed synergic effects of ECVs and DB + HA hydrogels on increasing the number of bone-specific cells and vessel and bone area compared to the control, DB + HA and ECV-treated groups.

**Conclusions:**

The DB enriched with ECVs can be an ideal scaffold for bone tissue engineering and may provide a suitable niche for bone cell migration and differentiation.

## Background

Bone defect repairing is one of the major challenges in regenerative medicine. Despite the high repair capability, spontaneous restoration of vast bone defects does not perform well for specific conditions such as trauma, fractures, and crushing [1–3]. The gold standard of bone defects treatment is autogenic transplantation, especially from iliac bones [4]; however, allogeneic and xenogenic grafts have also been used for bone regeneration. Despite the success of autologous bone transplantation, some limitations such as compliance of the transplanted bone shape in donor position, increase in operation time, resorption of the transplanted bone, and unavailable bone source, especially in the children [5] have created motivations for replacement materials. Engineered tissues, which are fabricated using suitable biocompatible materials and contain tissue-specific or stem cells, have the ability to recover bone biological functions [6].

One of the goals in bone tissue engineering is designing and fabricating absorbable and biodegradable scaffolds that can be replaced by newly-formed host bone to maintain tissue structural integrity over time [7]. A scaffold, as a temporary matrix provides a special environment for bone growth, and it will facilitate cell adhesion, migration and differentiation [8]. Different types of ceramic, polymer and composite scaffolds have been used for bone regenerative applications [9]. Recent studies have focused on the use of bioactive substances that stimulate cell migration to the site of the lesion. Since bone tissue contains inorganic and organic phases, the scaffolds for bone tissue engineering applications are usually fabricated from both mineral and organic biomaterials [10]. One of the most widely used minerals in bone scaffolds is hydroxyapatite (HA). HA constitutes approximately 60% of inorganic contents of dry bone and plays an important role in bone regeneration. HA is a resorbable bioactive mineral with osteoconductive and osteointegrative properties to promote bone repair. As HA is formed by ions commonly present in the extracellular matrix (ECM) and body fluids, it is highly biocompatible [11]. Although HA is considered as a useful biomaterial in bone tissue engineering, it has several limitations, including low mechanical properties and fragility [12]. Therefore, HA is used to fabricate bone scaffold in comparison with the other synthetic or natural biomaterials.

All tissues and organs contain a mixture of cells and non-cellular constituents that form a fully evolved network called ECM. ECM not only provides physical support for the cells, but also regulates many cellular processes including cell growth, migration, differentiation, survival and morphogenesis [13]. Collagen type I is the dominant organic component in bone ECM, while glycoproteins include osteocalcin, osteonectin, osteopontin, fibronectin and bone sialoprotein II as well as a wide range of growth factors such as bone morphogenetic proteins (BMPs) form non-collagenous organic components. Proteoglycans, including decorin, biglycan, lumican and osteoaderin, are also present in the bone matrix [10]. Luricin-rich proteoglycans play roles in cell proliferation as well as organic and mineral matrix deposition and remodeling. Glycosaminoglycans (GAG) contents of the bone interact with HA and prevents its degradation. They play some roles in cell–cell and cell–matrix interaction, bone morphogenesis and homeostasis [14]. Collagen and HA are responsible for mechanical strength of the bone. Osteoblast-matrix interaction is mediated by bone matrix proteins [10]. Collagen fibers are joined together by small proteoglycans and fragmented collagen strands. In addition to the structural and mechanical role in the adhesion of cells to the extracellular matrix, collagen plays roles in cell migration and differentiation. Remodeling of the ECM occurs during various physiological conditions, such as changes in the direction of the bone weight. Decellularized tissue provides biomimicry of the bone matrix that can be used for bone tissue engineering. Decellularized bone (DB) matrix from large animals has been demonstrated to have osteoinductive properties and promote osteogenesis of human mesenchymal stem cells [15]. Furthermore, DB and tricalcium phosphate, along with synthetic biomaterials, were used as bioink to fabricate scaffolds for bone regeneration [16].

Extracellular vesicles (ECV) are small vesicles that are released into the microenvironment surrounding the cells and are present in most of the tissue fluids [17]. Based on the size and biogenesis, ECVs are divided into three types: exosomes, microvesicular bodies, and apoptotic bodies [18]. These vesicles carry a selective set of receptor-ligands, enzymes, cytokines and genetic material from the mother cells. They subsequently bind and internalize into the target cells that lead to the sending of stimulatory or inhibitory messages, genetic reprogramming, and phenotypic alteration. Based on the sources, ECVs are also effective in physiological processes such as inflammation, regeneration angiogenesis [19] and waste disposal as well as pathological processes such as tumor progression and cardiovascular diseases [20]. Exosomes associated with the CNS play important roles in growth, remodeling, and communication between the glial and neuron cells. ECVs regulate the immune system and have a critical role in the immune response during both pathological and physiological processes [21]. ECVs have some regulatory impacts on bone-specific cells, and they release biologically active molecules to modulate bone functions, regeneration, and repair. ECVs originating from osteoblasts have been shown to promote bone marrow-derived mesenchymal stem cell (MSC) differentiation into osteoblast [17]. They also promote bone mineralization and regulate HA formation [22]. In addition, osteoblast-derived exosomes induce bone growth and regeneration by increasing vascular formation, and in fact, they stimulate endothelial cell proliferation and migration through NADH oxidase activation. ECVs isolated from osteocarcinoma cell line (MG63 cell line) enriched in metalloproteinases that facilitate matrix remodeling. Besides, EVCs from MG63 cell line have beneficial impact on mineralization [23]. With regards to the positive influence of osteoblast-derived ECVs, the current study was designed to find the effects of local administration of ECVs loaded in the scaffolds prepared by DB matrix and HA on the regeneration of mandibular bone defect model.

## Materials and methods

### Extraction of bone minerals

The diaphysis of the bovine femurs was boiled in tap water for 2–3 h, followed by washing with tap water to remove the undesirable tissues, including the bone marrow, tendons, and muscles. The bones were cut into small pieces, delipidated using acetone for 2 h, and washed several times with distilled water. To remove the organic matter, they were dried in 100 °C overnight, followed by incubating at 850 °C for 3 h. They were finally powdered by Balmill.

To evaluate the purity of the extracted hydroxyapatite, X-ray differentiation (XRD, Bruker D8 Advance) was performed, and the results were compared with the commercially prepared hydroxyapatite (Merck). After pulverizing, the samples were mounted on a holder. XRD was performed with = λ 1.54 A˚ (10˚-90˚ range) and Cu Kα as the radiation source. Besides, the particle size was evaluated by analyses of SEM images using imageJ software (https:// imagej.nih.gov/ij/index.html) or (NIH, Bethesda, MD).

## Decellularization of Bone Tissue

Sheep scapula was cut into 0.5 cm pieces, and after rinsing with phosphate buffer saline (PBS), they decalcified in 0.5 N HCl for 48 h. Then, they were incubated in 1% SDS for 48 h and rinsed with PBS to remove the trace of SDS from the tissue. All steps were performed at room temperature and on the stirrer**.**

## Evaluation of the decellularized tissues

Hematoxylin & Eosin and Hoechst staining were performed to evaluate the presence of nuclei in the tissues. To quantify DNA content, QIAamp® DNA Blood and Tissue Mini Kit (Qiagen GmbH, Hilden, Germany) was used. According to the manufacturer's instruction, 25 mg of lyophilized decellularized and intact bones was incubated in proteinase K at 56 °C until it was completely lysed. The samples were transferred to a microcentrifuge tube and washed by a buffer. DNA was eluted by adding ethanol and centrifuged in a mini spin column and extracted DNA quantified by spectrophotometer at γ = 260 nm, using the NanoDrop® ND-1000 (Nanodrop Technologies Inc., Wilmington, DE, USA)**.**

Histochemical staining was done to evaluate the retention of ECM content after decellularization. Alcian blue (pH 2.5), and PAS staining were performed to detect GAGs and neutral sugars, respectively. Collagen and elastic fiber retention was confirmed by Masson's trichrome and aldehyde fuchsin staining.

### Immunohistochemistry

Frozen sections at 5 µm thickness were prepared and fixed with 2.5% paraformaldehyde. The endogenous enzyme was blocked by incubating the samples in 3% H_2_O_2_ in methanol. Non-specific binding sites were blocked by incubating the samples in PBS containing 10% goat serum and 5% Bovine Serum Albumin (BSA). The samples were incubated in anti-collagen type I, -laminin and -fibronectin antibodies (All from Abcam PLC, Cambridge, MA, USA) at dilutions 1/250, 1/100 and 1/250, respectively. The sections were then incubated with horseradish peroxidase/streptavidin (1:10000; Abcam, USA) at room temperature for 20 min. Finally, diaminobenzidine was added as the chromogen.

Immunofluorescence was performed to detect osteopontin as well. To do this, frozen sections were fixed with 4% paraformaldehyde. Non-specific binding sites were blocked by incubating the samples in PBS containing 10% goat serum and 5% BSA. Then, the samples were incubated in anti-rabbit osteopontin primary antibody (1/100, Abcam, UK) overnight. The sections were incubated in Alexa flour 488-conjugated secondary antibody (ab150077) for 45 min and observed by fluorescence microscopy (Olympus, BX61).

### Scanning Electron Microscopy (SEM)

The ultrastructure of the decellularized matrix was evaluated by SEM. The samples were fixed with 2.5% glutaraldehyde for 20 min, dehydrated by gradually increasing concentration of ethanol, and dried in gradually increasing concentration of Hexamethyldisilazane (HMDS; Merck, Kenilworth, NJ, USA). A gold replica was prepared from each sample by Q150R- ES sputter coater (Quorum Technologies, London, UK), and micrographs were taken using a VEGA3 microscope (TESCAN, Brno, Czech Republic) at 10 kV accelerating voltage.

### Extraction of the extracellular vesicles from MG63 cell line

MG63 osteosarcoma cell line (purchase from Pasteur institute, Iran) were grown in RPMI-1640 culture medium (Bioadia) at 37 °C and 5% CO_2_ for 36–48 h, and then their supernatant was collected. ECVs were isolated from the culture medium by centrifugation at 300 *g* for 20 min. Then, the supernatant was filtered by 0.2 µm microfilter. The samples were then centrifuged at 100,000 *g* and 4 °C twice, each one for 90 min by an ultracentrifuge (Beckman, USA). The pellet was mixed with 100 µL PBS and stored at −70 °C until use.

### Characterization of extracellular vesicles

Transmission electron microscopy was used to evaluate the size and shape of the extracted ECVs. Flow cytometry was also performed to detect the surface markers on the ECV membrane. To do this, magnetic beads were vortexed 30 s, and then 20 µL of ECVs containing 16 µg was added to the magnetic beads and mixed on a shaker at 2–8 °C overnight. After washing with PBS, the tube was exposed to a magnetic field, the supernatant was removed, and 400 µL PBS was added. This stage was repeated twice. The ECV-bead complex was transferred to flow cytometry tubes and 20 µL of PE-conjugated anti-CD9, -CD63, and -CD81 antibodies (all from BD Pharmingen™) were added. ECVs were analyzed by FL2 channel of a flow cytometer (BD FACSCaliburTM, BD Biosciences). The data were analyzed by FlowJo software.

### Internalization of ECVs

MG63 cell line was cultured in the presence of RPMI containing 10% FBS (Gibco), 1% L-glutamine (Bioidea), and 1% penicillin/streptomycin (Gibco) at 37 °C and 5% CO_2_. ECVs were labeled with PKH26, using PKH26 red fluorescent cell linker kit (Sigma) according to the manufacturer's protocol. Briefly, 0.5 mL of ECVs mixed with 498 µL of dilution buffer and 2 µL PKH26 and incubated for 5 min and diluted with FBS at a ratio of 50/50. Then, the mixture was centrifuged at 100,000 *g* for 90 min at 4 °C. The pellets were mixed with 1 mL of the culture medium. Labeled ECVs were added to MG63 cell line cultures and incubated for 22 h. Finally, the internalization of the labeled ECVs was evaluated by fluorescence microscopy. For negative control, the same amount of dilution solution without ECVs was added to the cells.

### Dynamic Light Scattering (DLS)

ECV size was evaluated by nanoparticle analyzer (SZ-100 Horiba, Japon) equipped with a 532-nm wavelength, 10 mW power, and operating at an angle of 173. ECVs were transferred to cuvettes (ZEN0040, Malvern, Herrenberg, Germany). The measurements were made at a fixed position and at 25 °C. Three independent measurements were performed for each sample, and three samples were analyzed, and the mean value were calculated.

### Injectable hydrogel fabrication

Collagen extracted from the rat tails was lyophilized, and then 750 µL of 0.005% collagen was reconstituted with 125 µL of RPMI 10X. To neutralize acidity, 125 µL of the reconstructed buffer contained 2.2% sodium bicarbonate, and 4.8% HEPES (both from Sigma) was added. To prepared DB gel, a solution containing 1% ground DB in 3% acetic acid was stirred at 4 °C for three days, and then pepsin (20 U/g of DB dry weight) was added to dissolve the DB powder.

To prepare hydrogel, we mixed reconstituted tail rat collagen and solubilized DB at the ratio of 2:1, and then, 0.33 mg of hydroxyapatite powder per mL was added. Then, ECV at a concentration of 110 µg/mL was added to the collagen/DB mixture and incubated at 37 °C for thermogelation.

### In vitro study

MG63 cell line (1.5 × 10^4^ cell/well) was cultured on the 3D scaffolds along with 2D conventional culture conditions in the presence or absence of 100 µg ECVs at 37 °C and 5% CO_2_ for one, three, and seven days. The culture medium was RPMI-1640 containing 10% FBS, 1% L-glutamine, and 1% penicillin/streptomycin.

### Cytotoxicity assessment

To compare cell viability and proliferation in the hydrogel with/without ECVs, we replaced the culture media with 3-(4,5-dimethylthiazol-2-yl)-2,5-diphenyltetrazolium bromide (MTT, 0.5 mg/mL) for three hours on day one, three, and seven. Then, MTT was replaced by dimethyl sulfoxide, and the optical density of the eluted formazan was measured at 595 nm**.**

### Mineralization assessments

MG63 cell line cultured on 3D scaffolds and 2D conventional conditions with/without ECVs in the same condition as reported for cytotoxicity assay. After one, three, and seven days, the culture media were discarded, and the cell-containing scaffolds were washed with PBS, fixed with 75% ethanol for 20 min, and stained with 500 µL of 2% alizarin red S for 30 min. Then, the dye was eluted by incubating the cultures in 100 mM cetyl pyridinium chloride (Rad kimiagaran) for one hour. The concentration of the eluted dye was measured by spectroscopy at a wavelength of 405 nm**.**

Besides, the culture media were collected to assay the alkaline phosphates (ALP) activity. To evaluate the enzyme activity, a commercial kit (Parsazmoon, Iran), based on the capability of ALP to convert nitrophenyl to yellow nitrophenol, was used. The culture media were treated according to the manufacturer's instruction, and the intensity of yellow nitrophenol was measured using a microplate reader at 405 nm.

### Cell attachment and phenotype

To evaluate the cell attachment capability, 1.5 × 10^4^ cells were grown on both 3D scaffold, and polystyrene culture dish. After two hours, the amount of non-adherent cells was calculated and subtracted from the original cell number. To evaluate cell phenotype on the scaffolds, the same number of cells was seeded on a 3D scaffold with or without ECVs for two days and prepared for SEM in the same way as described above.

### In vivo studies

Forty male rabbits weighting 2.5–3 kg were purchased from the comparative animal research center, Shiraz, Iran. All animals were treated according to the ethics committee guidelines (IR.SUMS.REC.1397.277).

### Experimental design and surgery

Scaffolds were prepared as described for in vitro study. The same ratio of collagen, DB, HA, and ECVs was mixed to form an injectable solution for the experimental group. Forty rabbits were divided into four groups. Each group was divided into two subgroups; each one was followed up for either 2 or 8 weeks.

The animals were anesthetized by 50 mg/kg ketamine and 3.5 mg/kg xylazine. An incision was performed in the skin parallel to the base of the mandible, and masseteric muscle was exposed. While avoiding the facial artery, we created a defect size of 1 × 1 × 0.2 cm on the right mandible. The mandibular defects in the control and experimental groups were filled with 250 µL of collagen (control), collagen containing DB + HA + ECVs, collagen containing DB + HA, and collagen containing ECVs, respectively. The gel was inserted into the defect, and then the periosteum and skin were sutured, using Viryl 3–0 and Nylon 3–0 (Supa Medical Devices Co., Tehran, Iran).

After 2 and 8 weeks of follow up, the rabbits were killed and their mandibles were removed. X-ray radiography was performed with an X-ray machine (Planmeca Intra, Helsinki, Finland).

### Morphometrical analyses

The mandibles were fixed in 10% buffer formalin and decalcified in a decalcifying solution containing 4% HCl and 4% formic acid for 36 h. Paraffin-embedded samples were cut at 5 µm thickness and stained with H&E. Three sections from the first, middle, and end of each sample were chosen. Photographs were taken from systematic randomly selected fields, started from one edge of the grafted scaffold, and ended at the other edge. The number of osteoblasts, osteocytes, osteoclasts, and also the area occupied by the bone, connective tissue, adipose tissue, and vessels were estimated by the ImageJ software.

## Results

### XRD spectra of hydroxyapatite

**Fig. 1:**
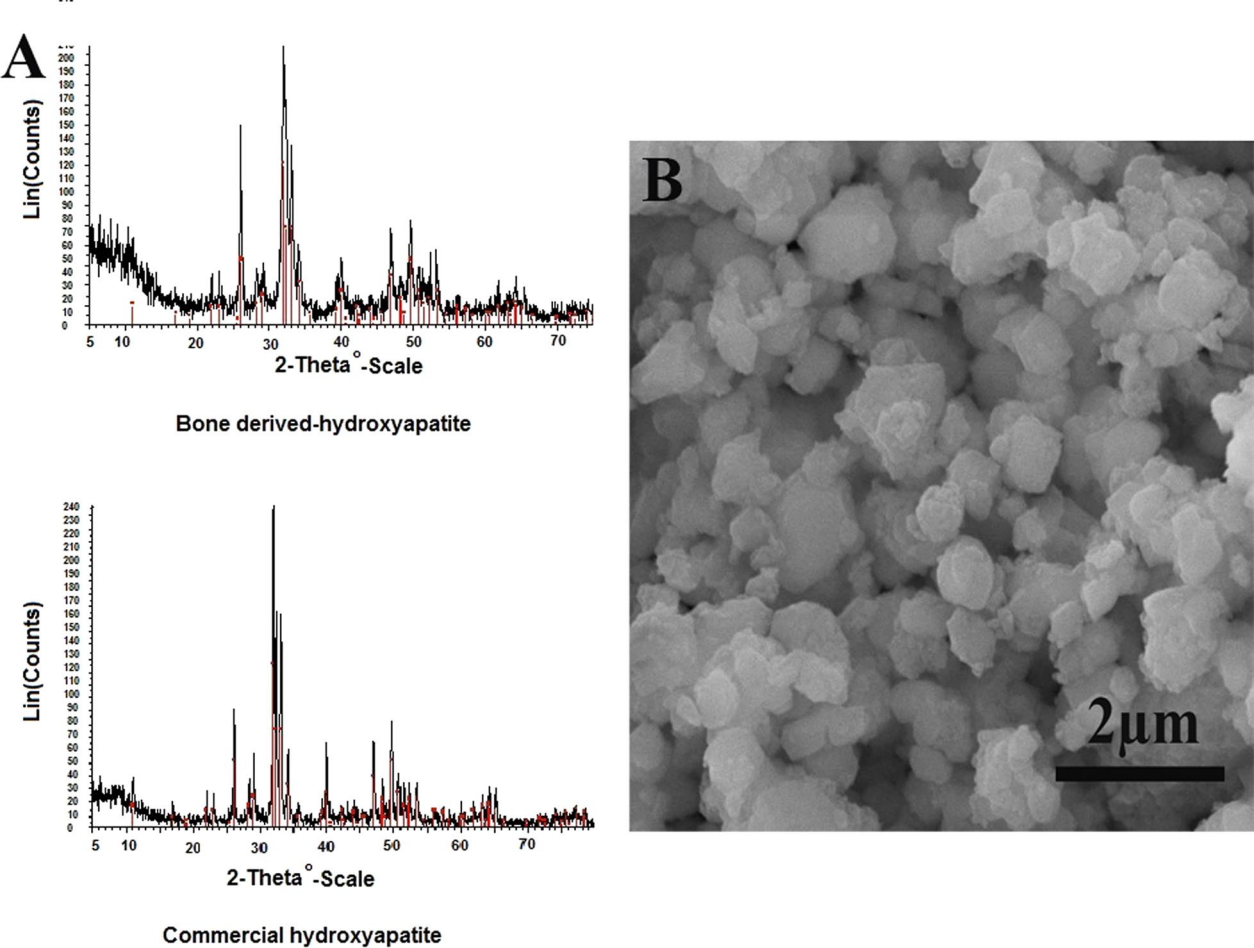
**a** Comparison of the HA extracted from the bovine bone and commercial HA. It indicates that most of the extracted inorganic components are HA. **b** Electron micrograph of extracted HA nanoparticles.

To confirm the purity, we compared the XRD spectra of the extracted inorganic components with commercially HA. As Fig. 1a demonstrates, crystalline peaks of the inorganic components derived from bovine bone are matched with that obtained from standard commercial HA. It indicates that most of the extracted inorganic components are HA. The particle size of HA was 430.2 ± 154.8 nm (Fig. 1b).

### Characterization of DB

**Fig. 2:**
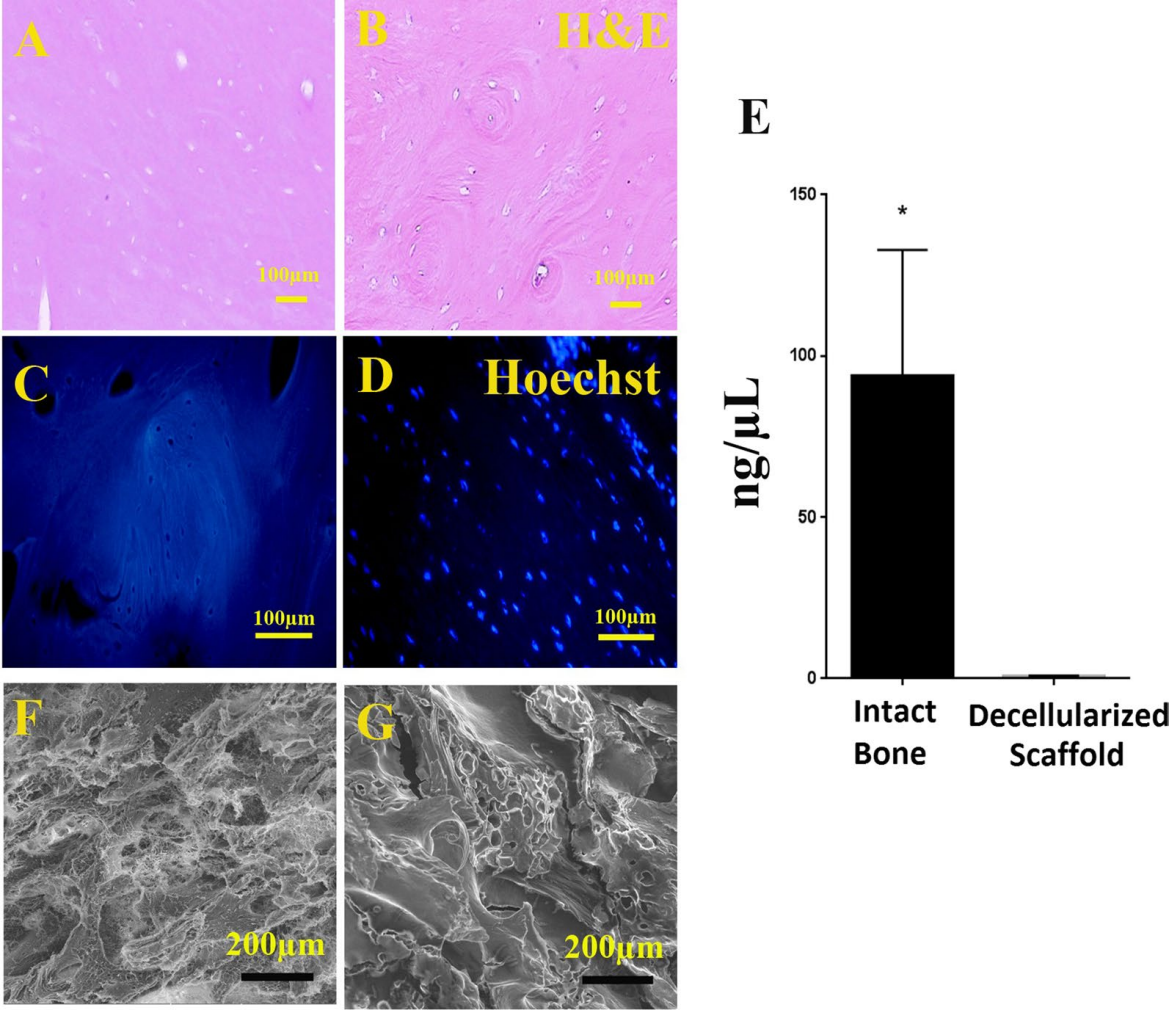
The sections of decellularized scaffolds (**a**, **c**) and intact bone (**b**, **d**) stained with Hoechst and H&E. Both staining confirm cell and nuclei depletion. Also, the graph (**e**) compares DNA content of decellularized scaffold and the intact bone. Scanning electron microphotographs revealed scaffold porosity, ultrastructure preservation and orientation of the collagen fibers of decellularized scaffold (**f**) and intact bone (**g**). * Significant difference with control group (P < 0.05)

To confirm the cell lysis and nuclei depletion, histological sections from DB were stained with Hoechst and H&E. Lacunae in DB were devoid of nuclei. Also, DNA quantification showed that not only the cell lysed, but also DNA remnants were washed out from the DB, so that the amount of DNA in the scaffold was lower than 50 ng/mg, which is less than the allowance amount (Fig. 2).

### ECM retention

SEM images also showed ultra-architecture preservation of the DB compared to intact bone (Fig. 2). The porosity and the orientation of the fibers in the DC scaffold were also similar to the intact sample.

**Fig. 3:**
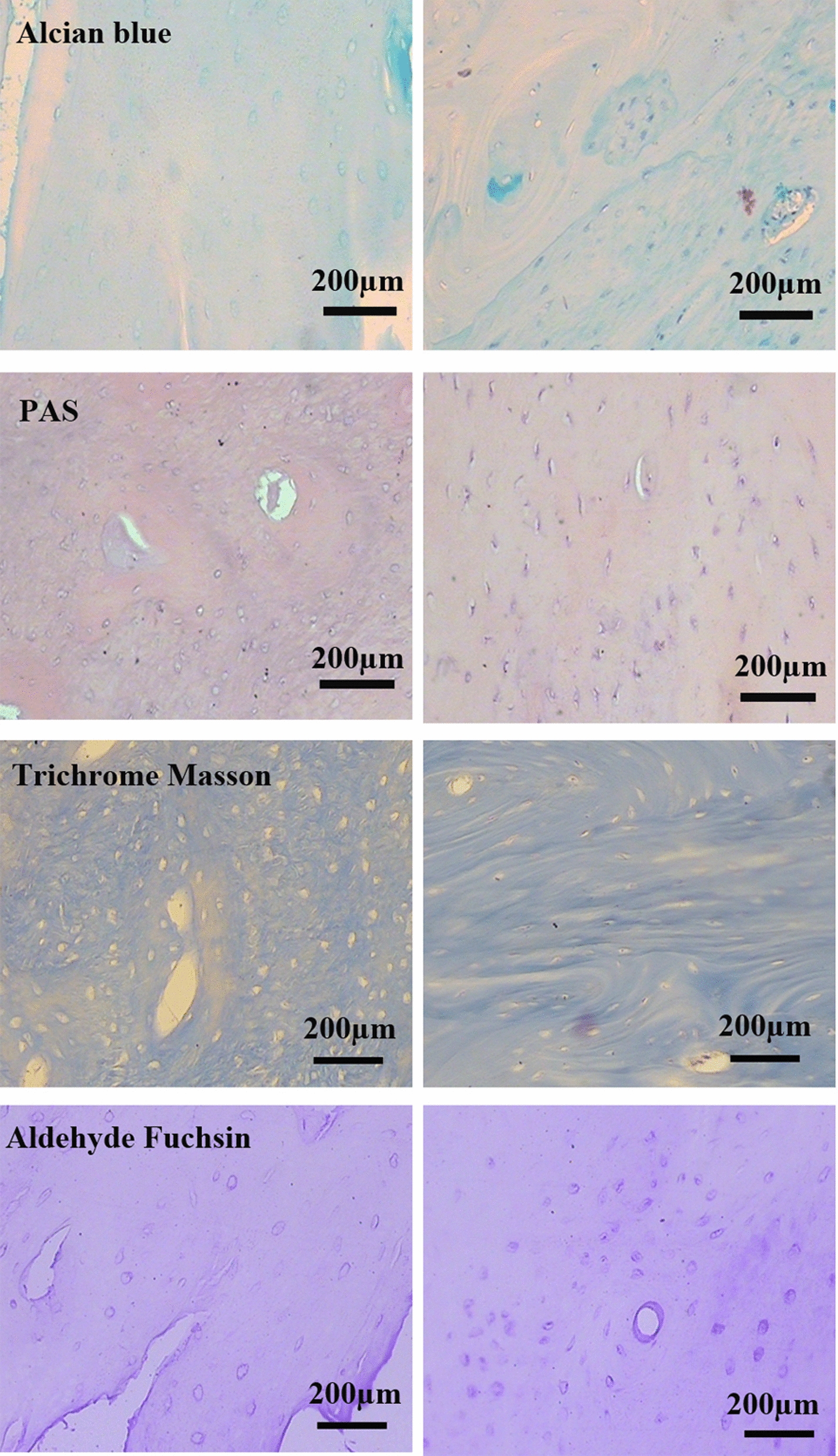
Histochemical Staining shows partially preservation of the ECM components after decellularization. Alcian blue (pH 2.5), aldehyde fuchsin and PAS stain acidic GAG and neutral carbohydrates preserved in ECM of decellularized and intact bone, and trichrome Masson stains collagen fibers

**Fig. 4:**
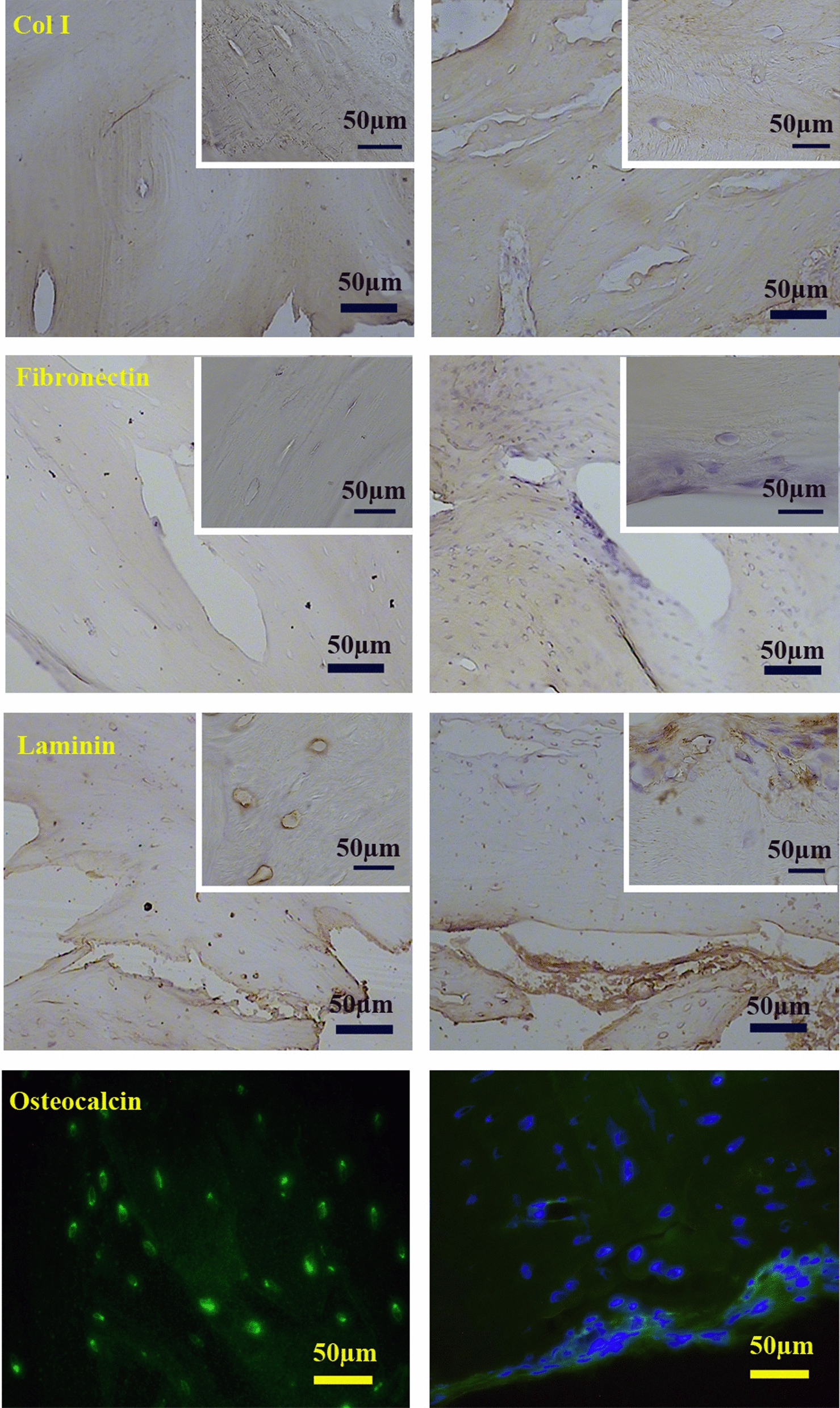
Immunohistochemistry shows preservation of collagen type I, fibronectin, laminin and osteopoitin in ECM of decellulsrized bone as compared to intact bone

H&E, trichrome Masson staining (Fig. 3), and immunohistochemistry for collagen type I (Fig. 4) showed partial preservation of the collagen fibers after decellularization. Alcian blue, aldehyde fuchsin, and PAS staining showed GAGs and neutral carbohydrates preserved in the ECM of DB; however, comparison of intact bone revealed partial washing of carbohydrate in the matrix surrounding lacuna (Fig. 3). Immunohistochemistry was also performed to evaluate the preservation of fibronectin, laminin, and osteopontin in ECM. The data indicated the retention of these glycoproteins in ECM after decellularization (Fig. 4).

### Extracellular vesicles characterization

**Fig. 5:**
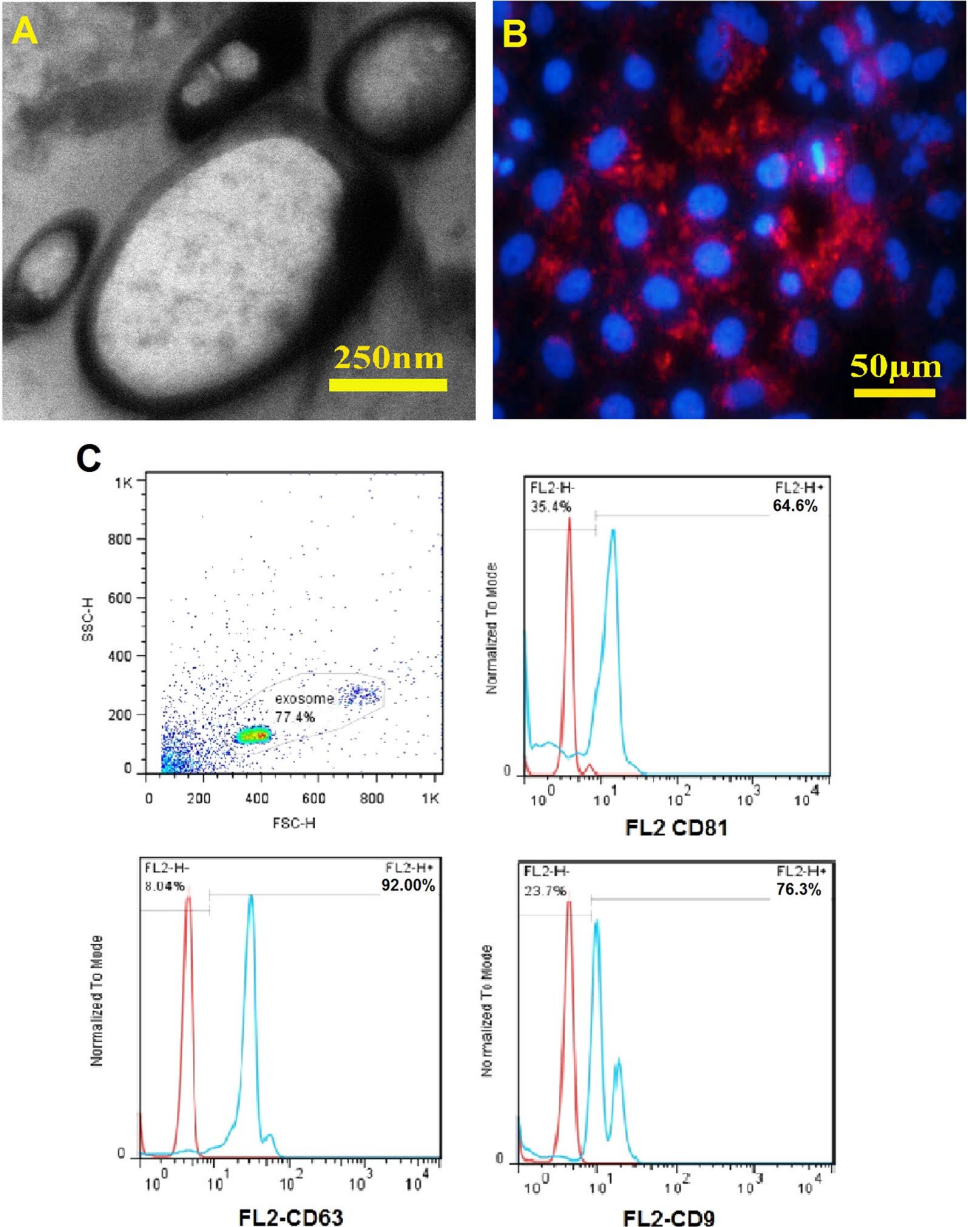
Transmission electron micrograph of the EVCs. **a** Shows the ultrastructure of the EVCs. Due to different sizes, EVC population is heterogeneous and contains exosome and microvesicles. Internalization potential of the extracellular vesicles exposed to osteoblasts (**b**). The red particle in the osteoblast indicates internalized ECVs. Also, flow cytometry of a representative sample of ECVs (**c**) shows that they were positive for CD_63_, CD_9_ and CD_81_

Figure 5 shows TEM photographs of ECVs with different sizes. They are membrane-bound particles containing a homogenous cytoplasm. Red particles in the osteoblast cell line indicate PKH-labeled ECVs internalization. It indicates that ECVs have the potential to transfer the signals into the target cells. Flow cytometry analyses showed that 92, 76.3 and 64.6% of the ECVs were positive for CD63, CD9, and CD81, respectively. The expression pattern of CD markers, as mentioned earlier, showed the heterogeneity in the ECV population (Fig. 5). DLS analyses also confirmed this heterogeneous feature as the size varies from 28.8–1331.8 nm.

### In vitro studies

#### Attachment test

**Fig. 6:**
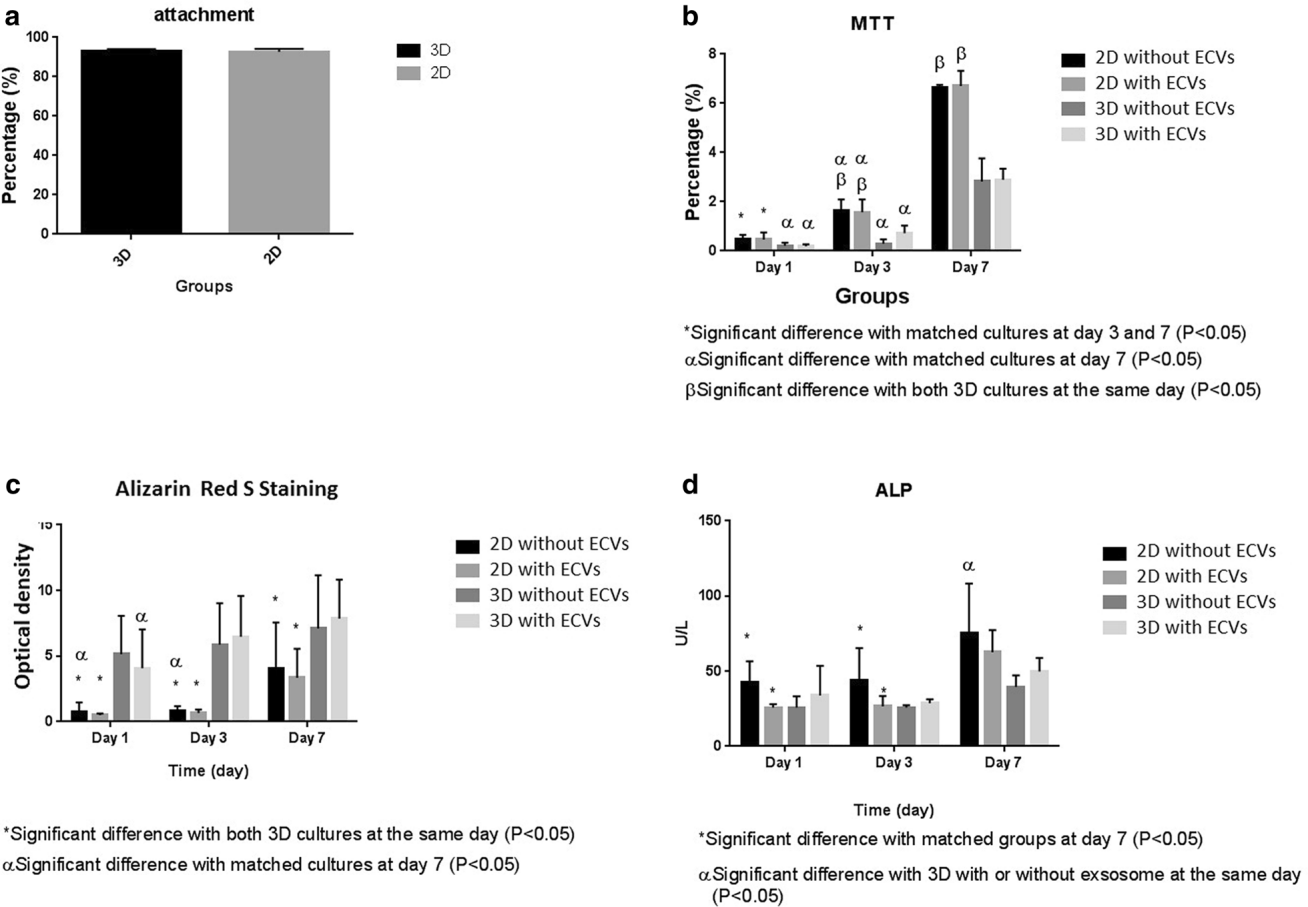
MG63 cell line showed similar attachment property to both decellularized scaffold and polystyrene culture dish (**a**). The graph compares viability of the cell on decellularized scaffold (**b**). It was revealed that the scaffolds were not toxic and protected cell proliferation, but the presence of ECVs had no influence on cell viability. Calcium deposition by alizarin red S staining (**c**) and ALP activity (**d**) increased as the time progress; however, the presence of ECVs did not affect on mineralization

The attachment test was performed to evaluate the adhesion capability of the cells to the scaffold. The data showed that the attachment property of the cells to the DC scaffold was similar to that on the polystyrene culture dish (Fig. 6a).

#### Viability test

2D culture system showed a significantly better condition for cell proliferation than 3D condition regardless of the presence or absence of ECVs after three and seven days (P < 0.01). A significant increase in viable cells in various conditions was recorded compared to the matched groups after days three and seven (P < 0.01) that indicated the scaffolds were not toxic and protected the cell proliferation. The presence of ECVs also did not influence cell viability (Fig. 6b).

### Mineralization assessments

Alizarin red S staining revealed that deposited calcium by MG63 cells increased significantly in both 3D culture conditions compared to 2D conventional culture system. 3D culture conditions accelerate the calcium deposition, so that a significant increase in calcium content was demonstrated at the early phase of the experiment (first day, 2D versus 3D with ECVs P = 0.003, 2D versus 3D without ECVs P = 0.004). However, as time progressed, the calcium deposition increased by the cells grown in 2D culture conditions as well. The presence of ECVs did not affect calcium deposition (Fig. 6c).

The data indicate that ALP activity was not influenced by the culture condition. The enzyme activity was increased significantly as the time progressed (2D without ECVs at day 1 versus at day 7, P = 0.011, 2D with ECVs at day 1 versus at day 7, P = 0.005). The presence of ECVs also had no influence on the enzyme activity. In the long term, 2D cultures provided a better condition for enzyme activity (2D without ECVs versus 3D P = 0.006), and it is in accordance with the data from cell viability assay as the higher number of cells in 2D condition produces the higher enzyme (Fig. 6d).

### In vivo studies

**Fig. 7:**
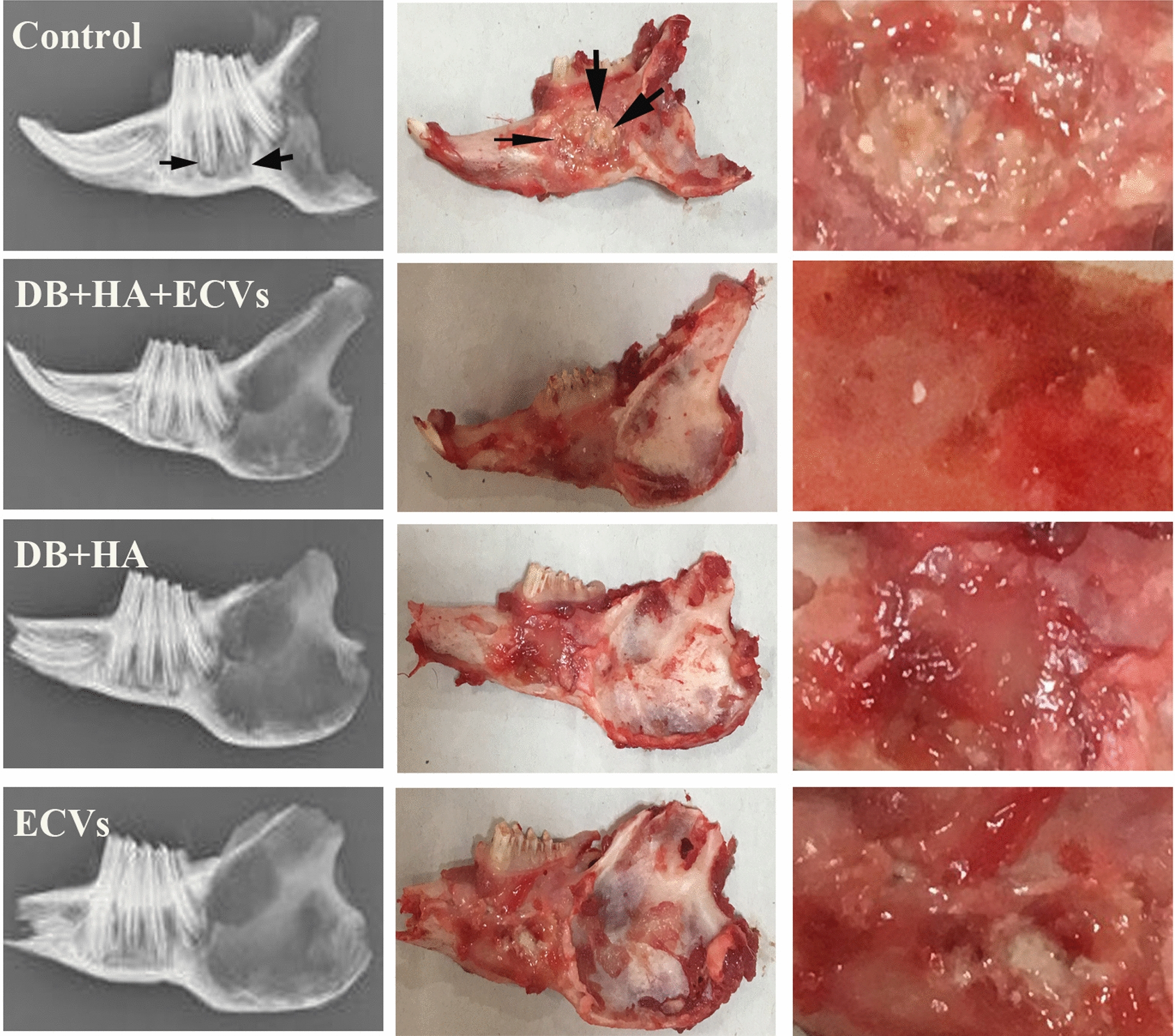
Comparing the gross and radiological examinations show that the repair accelerated in the defects treated with DB + HA + ECVs on 2 weeks after surgery. Arrows show the borders of the defects. *DB* decellularized bone, *HA* hydroxyapatite, *ECVs* extracellular vesicles

**Fig. 8:**
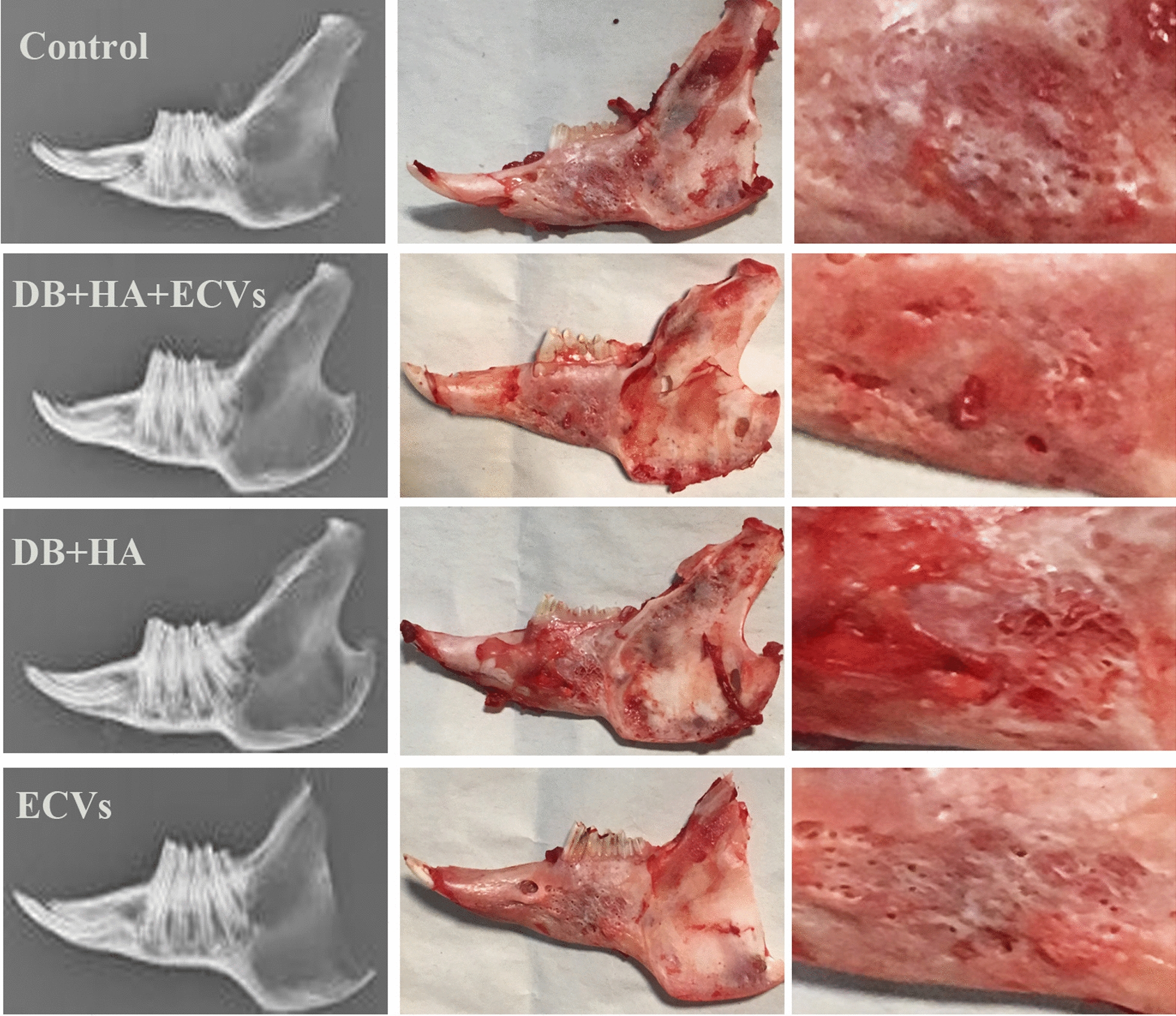
Comparing the gross and radiological examinations show that the repair in defects treated with DB + HA + ECVs was better than all other groups after 8 weeks. *DB* decellularized bone, *HA* hydroxyapatite, *ECVs* extracellular vesicles

**Fig. 9:**
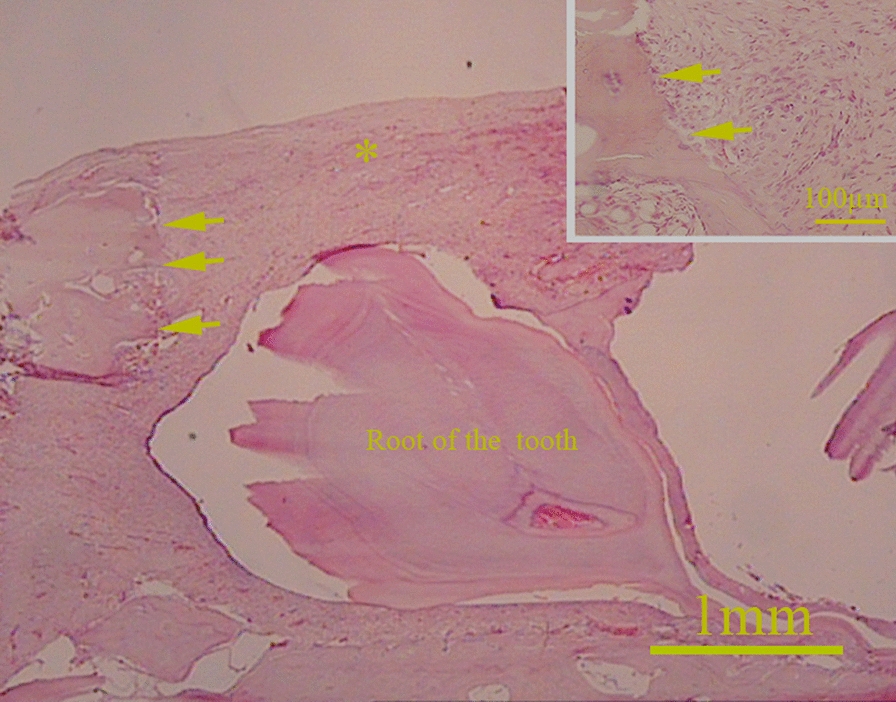
Low magnification of the interface between the host bone and implant (arrows) after 2 weeks. Small square shows higher magnification of this region. (*) shows the implant that replaced with newly formed repairing tissue. It contains small islands of bone spicules

Gross examination of the mandibles showed that the best repair happened in the defects treated with DB + HA + ECVs, and the worst belonged to control defects after both 2 and 8 weeks. The radiological examination also revealed that the bone density on the control group was less than all other groups (Figs. 7 and 8). Figure 9 shows interface between the host bone and implant after 2 weeks. It was replaced with connective tissue containing small islands of bone spicules.

**Fig. 10:**
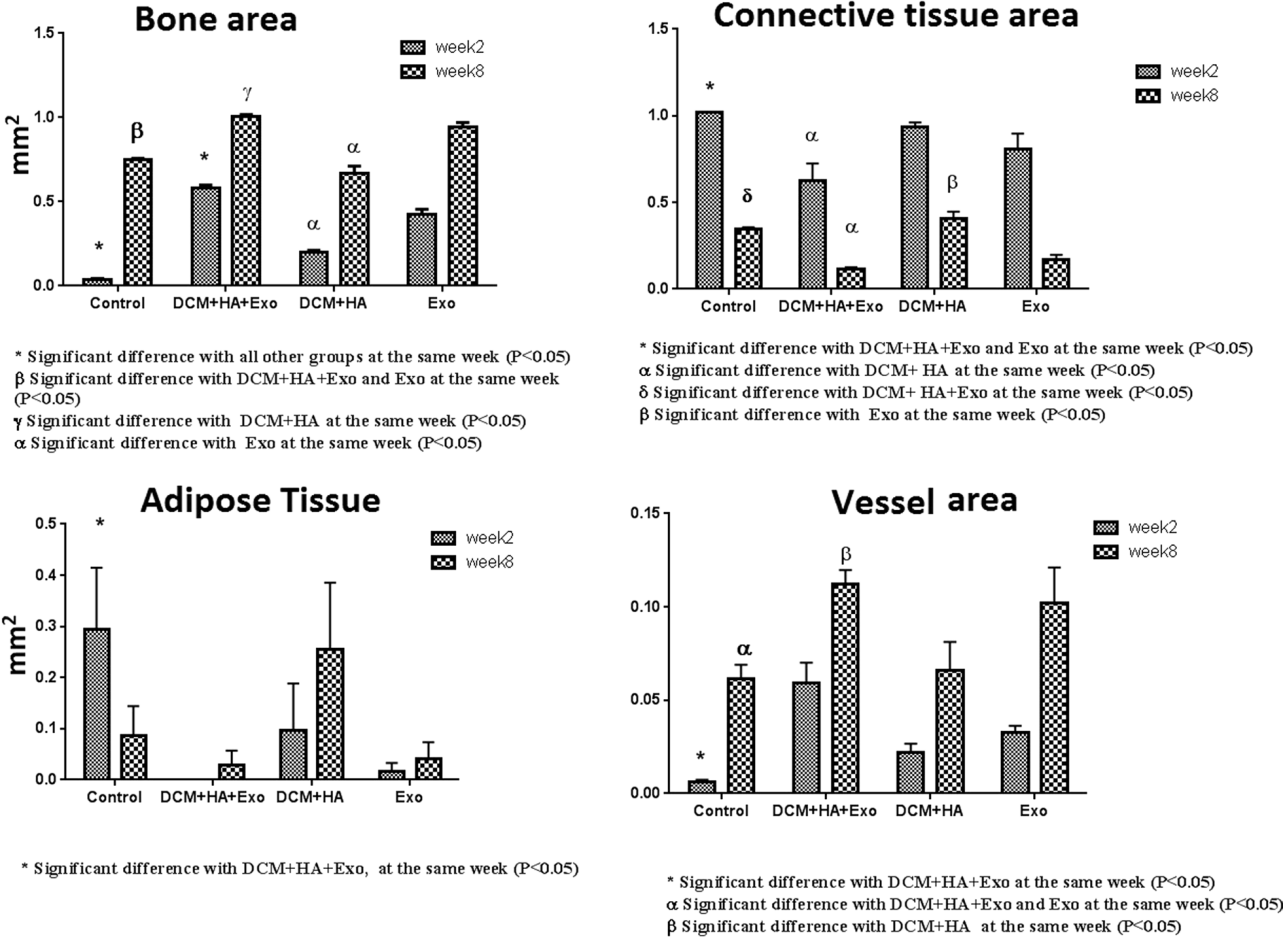
Morphometrical estimation of the bone area, fibrous connective tissue area, adipose tissue area and vessel area after 2 and 8 weeks. *DB* decellularized bone, *HA* hydroxyapatite, *ECVs* extracellular vesicles

At 2 weeks, in-vivo study revealed that the bone area increased significantly in all the treated groups compared to the control group (DB + HA + ECVs and ECVs versus control P < 0.0001 and DB + HA versus control P = 0.003). The best result belonged to the groups treated with DB + HA + ECVs, and the bone area in this group significantly increased compared to the groups which received DB + HA or ECVs alone (P < 0.0001 and P = 0.0047, respectively). This data indicates the synergic effects of DB + HA and ECVs in bone regeneration. ECV treatment also increased the bone area compared to that in the DB + HA-treated group (P = 0.0002). Eight weeks after the surgery, the bone area was significantly higher in the groups treated with DB + HA + ECVs and ECVs compared to the control (P < 0.0001, P = 0.0008, respectively) and DB + HA groups (for both P < 0.0001, Fig. 10a).

The morphometrical estimations demonstrated that both groups treated with ECVs (with or without DB + HA) contained lower areas occupied by fibrous connective tissue compared with control group (P < 0.0001 and P = 0.0318, respectively) and 8 weeks (P = 0.0172) after recovery (Fig. 10b).

After 2 weeks of recovery, the migrating cells into the DB + HA + ECVs-treated defect showed the lowest potential to differentiate into adipocytes compared to control group (P = 0.0446), while DB + HA + ECVs- treated defects showed the least adipose tissue area. After 8 weeks, the area occupied by adipose tissue was similar in all groups (Fig. 10c).

Although all treated groups induced the angiogenesis compared to the control group, the defects treated with DB + HA + ECVs contained a significant increase in the vessel area respect to control defect after 2 weeks recovery (P = 0.0055). After 8 weeks, two defects received DB + HA + ECVs and ECVs containing significantly higher vessel area compared to control groups (P = 0.008, P = 0.0424, respectively). Also, ECVs-treated group contained significant higher vessel area compared to DB + HA-treated group (P = 0.0178, Fig. 10d).

**Fig. 11:**
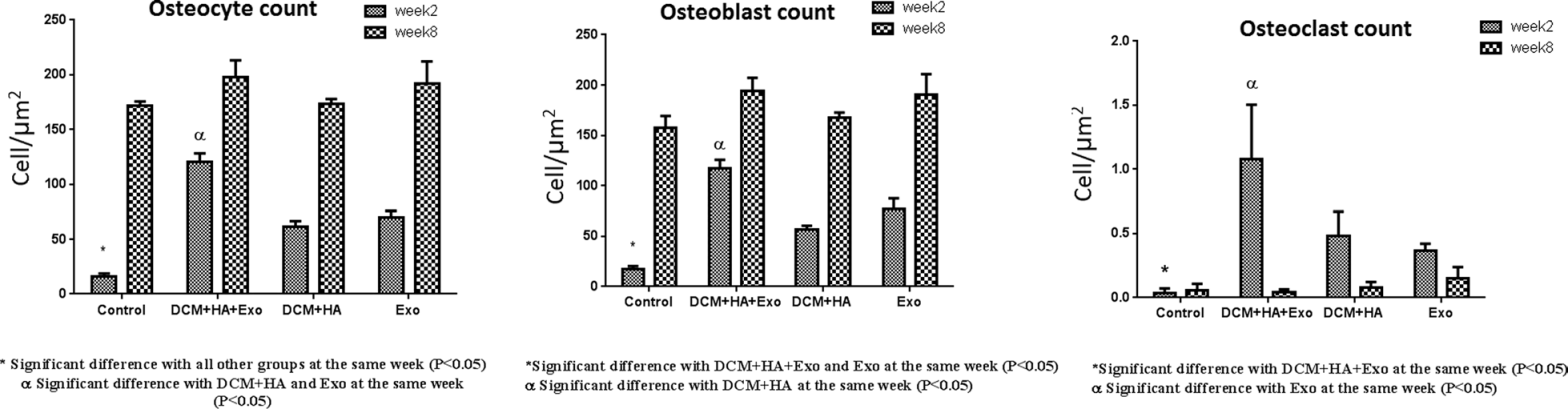
Morphometrical estimation of the number of osteocytes, osteoblasts and osteoclasts after 2 and 8 weeks recovery. *DB* decellularized bone, *HA* hydroxyapatite, *ECVs* extracellular vesicles

The estimation of the osteocyte number revealed that all treated groups contained significantly higher number of cells compared to the control group (P < 0.0001, P = 0.0155, P = 0.0035, respectively); however, the defects treated with DB + HA + ECVs showed significantly highest number of osteocytes after 2 weeks (Fig. 11a). At the same time, the defects treated with DB + HA + ECVs and ECVs contained significant higher number of osteoblasts compared to control defects (P < 0.0001 and P = 0.0039, respectively, Fig. 11b).

**Fig. 12:**
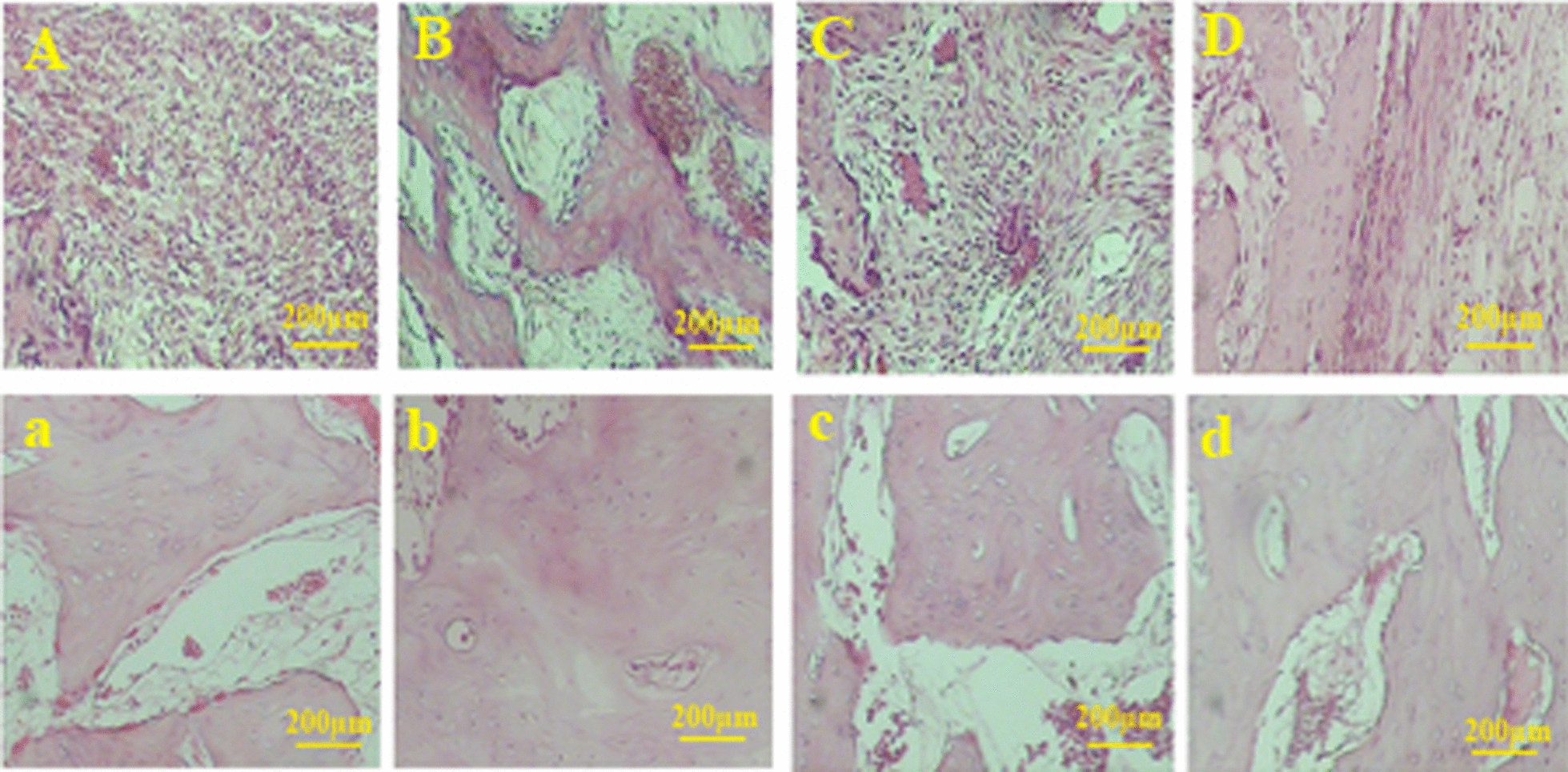
Histological sections of repairing defects treated with various scaffolds after 2 weeks recovery. The control defect (**A**,** a**) and the defects treated with DB + HA + ECVs (**B**,** b**), DB + HA (**C**,** c**) and ECVs (**D**,** d**) after 2 (above) and 8 (below) weeks. *DB* decellularized bone, *HA* hydroxyapatite, *ECVs* extracellular vesicles

**Fig. 13:**
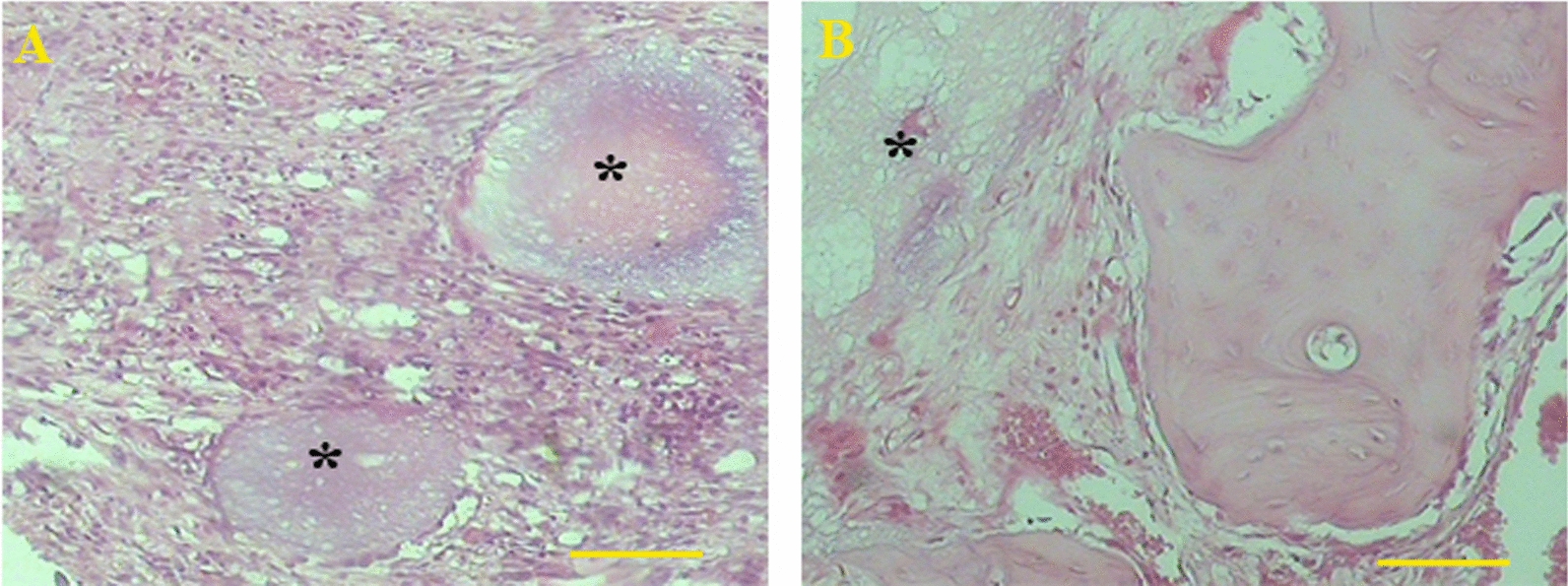
The remnant of the scaffolds (*) in the defect after 2 (**a**) and 8 (**b**) weeks

After 8 week recovery, the number of osteoblasts and osteocytes was statistically similar in all groups. The number of osteoclasts was also higher in all treated groups compared to the control cultures; however, the significant difference was detected only for the defects treated with DB + HA + ECVs (P = 0.0009). After 8 weeks, the number of osteoclasts was similar in all groups (Fig. 11c). Figure 12 shows the histological sections from repairing defects treated with various scaffolds at 2 and 8 weeks, respectively. In some cases, the remnant of scaffolds can be observed in the sections. After 2 weeks recovery, the percentages of the samples contained remnant scaffolds are 3.3, 11.6, 3.3 and 6.6% for DB + HA + ECV, DB + HA, ECV and control groups, respectively. After 8 weeks, the remnant of scaffolds was observed in 3.3% samples in DB + HA-treated group (Fig. 13).

## Discussion

ECM of the scaffold contains several bioactive substances including fibronectin, collagen, and different types of growth factors [24]. The purpose of decellularization is clearance of antigenic cell debris and provides a suitable scaffold from tissue–specific ECM, so that appropriate cells can be cultured on them. A recent research has also suggested that the constituents and architecture of the ECM provide a “zip codes” for cells. This zip code guides and supports the phenotype, attachment, and differentiation of the cells [25]. In the present study, we showed that decellularized scaffolds retained the ECM contents such as osteopontin, laminin, fibronectin, GAGs, and collagen. These components have many roles in bone repair. For instance, it has been shown that osteopontin contributes to cell adhesion and accelerates mineralization. The mineralization is critical for the integration of the newly formed bone with the healthy bone surrounding the defect [26]. Also, osteoblasts cultured on laminin-coated titanium showed higher ALP activity than the uncoated one [27]. Laminin also has several roles in cell migration, survival, attachment, and promotes osteogenic differentiation [28]. Collagen I, GAG, fibronectin [29] and HA [30] also promote the bone repair and remodeling. We showed that the number of osteocytes and bone area increased significantly in the defect treated with DC + HA scaffold compared to the untreated control defect. It indicates that the components in the decellularized ECM, along with HA, have osteoinductive and osteoconductive properties. The demineralized decellularized scaffold was also used for bone tissue engineering. Both in vitro [31] and in vivo [15] studies showed that decellularized scaffolds had an osteoinductive effect, and it has a beneficial influence on bone-specific marker expression and calcium deposition [31]. Besides, in vivo tests showed a significantly higher number of osteoblast and bone areas in the defect filled with DC + HA compared to the control condition**.**

MTT test showed that cell proliferation decreased in 3D conditions. As the review article revealed, culturing on 3D scaffolds recapitulate the in vivo condition, and as a result, the cell proliferation is limited when compared to 2D condition [31]. In a study, MSCs were grown on a decalcified bone loaded with MSC-derived ECVs. Along with our findings, it was detected that the presence of ECVs did not influence the cell proliferation [32]. However, ECVs from different sources have different macromolecular components and surface markers and exert different effects on bone activity.

In the current study, the in vitro test revealed that the cells cultured on DC + HA scaffolds deposited more calcium without any toxicity compared to 2D conventional culture condition. We also found that the ALP activity was higher in long term 2D conditions compared to 3D condition. Since cells proliferate with a higher rate in 2D than 3D condition, the cell population increases in 2D cultures as the time progresses. Because the ALP concentration has a positive correlation twith the number of osteoblasts [33], the enzyme activity is higher in 2D conditions as well. ECVs can stimulate the bone regeneration and directly regulate the osteoblast activity and proliferation [34]. Osteoblast-derived exosomes have been shown to induce MSC differentiation toward osteoblasts [35]. The current study revealed that in vivo administration of ECVs in DC + HA scaffolds led to a significant increase in the osteoblast number. The presence of RANKL has been reported on the surface of osteoblast-derived ECVs. RANKL interacts with RANK on the surface of osteoclast precursors and leads to osteoclast differentiation [35]. Osteoclasts, in turn, can regulate the osteoblast activity and bone formation [36]. This can explain why we found the positive influence of ECVs on the osteoblast number and bone formation in vivo, but in vitro adding of ECVs to both 2D and 3D conditions does not affect the osteoblast activity.

In vivo study showed that intraperitoneal injection of osteoblast-derived ECVs to the RANKL^−/−^ transgenic mouse exhibited osteoclastogenic potential [37]. The data from the current study also confirmed that ECVs in the DC + HA environment could increase the osteoclast numbers.

Decalcified bone coated with bone marrow MSC-derived ECVs were grafted subcutaneously in nude mouse, and it was found that ECV loading had no impact on bone formation [32]. In contrast to this study, we found that both ECVs-treated conditions accelerated the osteoblast and osteocyte differentiation and increased the bone formation. These contradictory results may attribute to the various macromolecule components and surface markers present in ECVs derived from different sources.

Angiogenesis is a critical process during bone healing, and endothelial cell migration to the repairing or engineered bone has a positive effect on regeneration. ECVs promote endothelial cell proliferation and migration and cause the cells to form vascular tubes. In vivo treatment of MSC-derived ECVs also stimulates angiogenesis in different tissues [34] including transplanted decalcified bone [32]. Administration of the autologous bone marrow MSC-derived exosome in calvaria defect induced bone repair and angiogenesis [38].

The data from the present study also confirmed that osteoblast-derived ECVs increased the surface area occupied by vascular components. This may indicate that the loaded ECVs have angiogenic activity.

The administration of DC + HA scaffold with ECVs showed a synergistic effect, so that some parameters such as the number of osteoblasts, osteocytes, osteoclasts, bone, and vessel area increased in the treated defect. Also, DC + HA can be considered as an excellent vehicle to introduce ECVs for bone regeneration. Besides, the appropriate degradation rate of DC + HA may decelerate ECV releasing rate, so that the long term effect of ECVs can also be observed in the defect after 8 weeks.

HA alone or in combination with the other biomaterials was used to accelerate repairing the mandibular defect in animal model [39]. HA with different particle size were used to treat the defects [39, 40]. HA remains for long term on the histological sections, when large particle size was used [39], while using HA nanoparticles for treatment could not be observed within first weeks after transplantation [40, 41].

## Limitations of the study

The current study encountered several limitations such as quantification of mineralization in the newly formed bone treated with engineered tissue. The other limitation is comparing newly formed matrix content with intact bone.

## Conclusion

In vivo study revealed positive effects of DB + HA hydrogel on bone repair. Since in vitro study indicated EVCs could not improve the osteoblast functions, in vivo beneficial effects of ECVs might attribute to their indirect influence on the osteoblasts. Synergistic effects of DB + HA and ECVs led to an increase in the bone area and the number of bone-specific cells as well as angiogenesis.

## Data Availability

All data generated or analyzed during this study are included in this published article.

## Abbreviations

ALP: Alkaline Phosphates
BMPs: Bone Morphogenetic Proteins
BSA: Bovine Serum Albumin
DB: Decellularized Bone
DLS: Dynamic Light Scattering
ECV: Extracellular vesicles
ECM: Bone Extracellular Matrix
HA: Hydroxyapatite
H&E: Hematoxylin & Eosin
HEPES: 4-(2-Hydroxyethyl)-1-piperazineethanesulfonic acid
GAG: Glycosaminoglycans
MSC: Mesenchymal Stem Cell
MTT: 3-(4,5-Dimethylthiazol-2-yl)-2,5-diphenyltetrazolium bromide
NADH: Nicotinamide adenine dinucleotide
PBS: Phosphate Buffer Saline
PAS: Periodic Acid Schiff
SEM: Scanning Electron Microscopy
SDS: Sodium dodecyl sulfate
XRD: X-ray Power diffraction
